# PERK Limits *Drosophila* Lifespan by Promoting Intestinal Stem Cell Proliferation in Response to ER Stress

**DOI:** 10.1371/journal.pgen.1005220

**Published:** 2015-05-06

**Authors:** Lifen Wang, Hyung Don Ryoo, Yanyan Qi, Heinrich Jasper

**Affiliations:** 1 Buck Institute for Research on Aging, Novato, California, United States of America; 2 Department of Cell Biology, New York University School of Medicine, New York, New York, United States of America; The University of North Carolina at Chapel Hill, UNITED STATES

## Abstract

Intestinal homeostasis requires precise control of intestinal stem cell (ISC) proliferation. In *Drosophila*, this control declines with age largely due to chronic activation of stress signaling and associated chronic inflammatory conditions. An important contributor to this condition is the age-associated increase in endoplasmic reticulum (ER) stress. Here we show that the PKR-like ER kinase (PERK) integrates both cell-autonomous and non-autonomous ER stress stimuli to induce ISC proliferation. In addition to responding to cell-intrinsic ER stress, PERK is also specifically activated in ISCs by JAK/Stat signaling in response to ER stress in neighboring cells. The activation of PERK is required for homeostatic regeneration, as well as for acute regenerative responses, yet the chronic engagement of this response becomes deleterious in aging flies. Accordingly, knocking down PERK in ISCs is sufficient to promote intestinal homeostasis and extend lifespan. Our studies highlight the significance of the PERK branch of the unfolded protein response of the ER (UPR^ER^) in intestinal homeostasis and provide a viable strategy to improve organismal health- and lifespan.

## Author Summary

The long-term maintenance of tissue homeostasis in barrier epithelia requires precise coordination of cellular stress and inflammatory responses with regenerative processes. This coordination is lost with age, resulting in degenerative and proliferative diseases. The Unfolded Protein Response of the Endoplasmic Reticulum (UPR^ER^) is emerging as a central regulator of tissue homeostasis in barrier epithelia. The UPR^ER^ adjusts the protein folding capacity of the ER in response to protein stress in stem cells and differentiated cells, and thus influences proliferative homeostasis, cell differentiation and epithelial inflammatory responses. How these responses are coordinated to maintain epithelial homeostasis in aging organisms remains unclear. In a previous study, we have found that the UPR^ER^ controls intestinal stem cell (ISC) proliferation in the *Drosophila* intestinal epithelium by influencing the intracellular redox state. How signaling through the canonical ER stress sensor PERK (PKR-like ER kinase) is integrated into this signaling network remained unclear. Here we show that PERK serves as a central regulator of ISC proliferation and tissue homeostasis in response ER stress. Strikingly, we find that within the intestinal epithelium, PERK is activated specifically in ISCs in response to both systemic and local ER stress, and is required for ISC proliferation under both homeostatic and stress conditions. We identify JAK/Stat signaling as an activator of PERK in ISCs in response to ER stress in neighboring cells, and find that the wide-spread age-associated increase in PERK activity in ISCs is a cause of age-related dysplasia in this tissue. Accordingly, limiting PERK activity in ISCs promotes homeostasis of the intestinal epithelium in old flies and extends lifespan.

## Introduction

Progressive decline of proliferative homeostasis in high-turnover tissues is a hallmark of aging, resulting in cancers and degenerative diseases. This is of particular relevance in barrier epithelia, such as the intestinal epithelium, where homeostatic tissue renewal has to be balanced with acute regenerative episodes in response to acute damage or infection. Accordingly, the control of intestinal stem cell (ISC) proliferation has to integrate endogenous control mechanisms with stress and inflammatory signals that promote mitogenic activity of these cells. How cellular stress responses of intestinal epithelial cells (IECs) and intestinal stem cells (ISCs) coordinate and maintain such regenerative processes is a critical question that will provide insight into the etiology of pathologies ranging from inflammatory bowel diseases (IBDs) to colorectal cancers.

Long-term homeostasis of the intestinal epithelium is significantly impacted by ER stress. In mouse models for IBDs, ER stress is increased in the intestinal epithelium [1–3], and genetic conditions that impair protein folding capacity in the ER of IECs result in complex cell-autonomous and non-autonomous activation of stress signaling pathways, triggering inflammatory conditions similar to IBDs [4–10]. Recent studies in mice suggest that the UPR^ER^ may also influence regenerative processes in the gut directly, as it is engaged in cells transitioning from a stem-like state into the transit amplifying state in the small intestine of mice [11]. In flies, ER stress promotes ISC proliferation, and increased ER stress across the intestinal epithelium is associated with age-related dysplasia in this tissue [1–3,12]. The downstream signaling mechanisms promoting ISC proliferation in response to ER stress remain unclear.

### Autonomous and non-autonomous responses to ER stress

**Fig 1:**
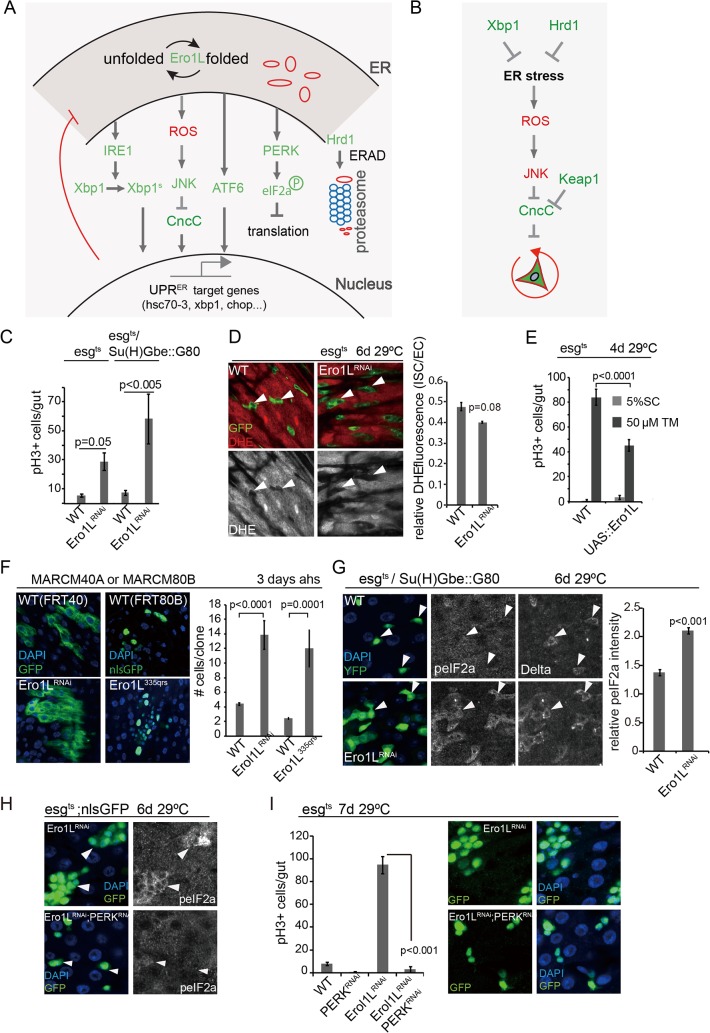
ROS-independent induction of proliferation of *ero1l*-deficient ISCs. (A)Major branches of UPR^ER^ and ROS signaling pathways in *Drosophila*. (B)Model of Xbp1-Hrd1 branch of the UPR^ER^ and ROS signaling network regulating ISC proliferation. (C)Loss of Ero1L in ISCs promotes ISC proliferation. PH3+ cell frequency in intestines is quantified for wild-type flies and flies expressing Ero1L^RNAi^ both in ISCs/EBs (esg^ts^) and specifically in ISCs (esg^ts^,Su(H)Gbe::G80). Averages and SEM are shown. P values from Student’s T test, N>15. (D)DHE fluorescence in wild-type controls and Ero1L^RNAi^ expressing ISCs/EBs (esg^ts^). GFP, green; DHE, red; DHE is shown as separate channel in white. Averages and SEM for relative DHE intensity are shown. P values from Student’s T test, N > 200 (from 5–8 guts). (E)Quantification of pH3^+^ cells in wild-type flies and in flies over-expressing Ero1L under the control of esg::Gal4, UAS-GFP; tubG80^ts^ exposed to mock (5% sucrose) and tunicamycin. Averages and SEM are shown. P values from Student’s Test. N = 10. (F)Larger clone sizes (MARCM) in Ero1LRNAi or Ero1L^335QRS^ condition compared with their own wild-type controls at 3 day after heat shock. Averages and SEM are shown. P values from Student’s T test. Number of clones analyzed: n = 121 (FRT40); n = 89 (Ero1LRNAi); n = 66 (FRT80B); n = 63 (Ero1L^335QRS^). (G)eIF2α phosphorylation in Ero1L-deficient ISCs (knockdown using esg::Gal4, Su(H)Gbe::G80, tubG80ts). DAPI, blue; YFP, green; peIF2α or Dl shown as separate channels in white. Arrowheads for orientation. Quantification of phospho-eIF2α staining in ISCs relative to neighboring ECs in wild-type guts and in guts with Ero1L-deficient ISCs shown on the right. Averages and SEM are shown. P values from Student’s Test. (H)Increased eIF2α phosphorylation in Ero1L-deficient ISCs/EBs (esg^ts^) is repressed by loss of PERK in ISCs/EBs (esg^ts^). DAPI, blue; nlsGFP, green; peIF2α, white. Arrowheads for orientation. (I)Knockdown of PERK in ISCs/EBs (esg^ts^) inhibits ISCs proliferation induced by loss of *Ero1L*. Frequency of pH3+ cells quantified for wild-type flies and flies expressing dsRNA against PERK only,dsRNA against Ero1L only, and for flies co-expressing dsRNA against PERK and dsRNA against Ero1L (combined with Dicer2 to enhance RNAi efficiency; shown on the left). Averages and SEM are shown. P values from Student’s T test, N = 10. Representative images are shown on the right. DAPI, blue; nlsGFP, green. See also S1 Fig.

Three highly conserved UPR^ER^ sensors coordinate the cell-autonomous response to ER stress: PERK, the transcription factor ATF6, and the endoribonuclease IRE1 (Fig 1B) [4–10,13]. IRE1 promotes splicing of the mRNA encoding the transcription factor Xbp1, PERK phosphorylates and inhibits the translation initiation factor 2 alpha (eIF2α) [11,14,15], and ER stress-induced cleavage of ATF6 promotes its nuclear translocation and activation of stress response genes, including Xbp1 [16]. The activation of Xbp1 and ATF6 results in transcriptional induction of ER chaperones, of genes encoding ER components, and of factors required to degrade un/misfolded proteins through ER-associated degradation (ERAD), thus enhancing ER folding capacity and proteostatic tolerance [17–19].

Phosphorylation of eIF2α, in turn, results in a broad, but selective decrease in protein translation, reducing the protein load in the ER, but also allowing selective translation of transcripts that contain alternative upstream open reading frames (uORFs), including the transcription factor ATF4. ATF4 target genes promote ER stress tolerance and boost antioxidant defenses [20–22]. PERK further phosphorylates and activates Nrf2 (nuclear factor-erythroid-derived 2 (NF-E2)-related factor 2), a central regulator of anti-oxidant gene expression [23,24].

Studies in worms have shown that, in addition to these cell-autonomous responses to ER stress, local activation of the UPR^ER^ can trigger UPR^ER^ responses in distant tissues, indicating that endocrine processes exist that coordinate such stress responses across cells and tissues [25–28]. The mechanism(s) regulating and mediating these non-autonomous responses remain elusive.

### Coordination of ER stress and oxidative stress responses in stem cells

By regulating eIF2α, ATF4 and Nrf2, PERK activation integrates the response to both protein misfolding in the ER and to misfolding-associated oxidative stress. Accumulation of un/misfolded proteins in the ER results in the production of reactive oxygen species (ROS), most likely due to the generation of hydrogen peroxide as a byproduct of protein disulfide bond formation by protein disulfide isomerase (PDI) and ER oxidoreductin 1 (Ero1) [29–31].

The coordinated control of cellular protein and redox homeostasis by the UPR^ER^ and other stress signaling pathways is likely critical to maintain SC function, as the intracellular redox state significantly impacts SC pluripotency, proliferative activity, and differentiation [32–35]. We have recently shown that this coordination is achieved in *Drosophila* ISCs by integration of Nrf2/CncC-mediated responses and Xbp1-mediated ER stress responses [12]. The fly orthologue of Nrf2, CncC, counteracts intracellular oxidants and limits proliferative activity of ISCs [34]. In ISCs, CncC is inhibited in response to high ER stress (as in Xbp1 loss-of-function conditions), resulting in increased oxidative stress and activation of ISC proliferation [12,34].

### Control of epithelial regeneration by *Drosophila* intestinal stem cells

The *Drosophila* ISC lineage exhibits a high degree of functional and morphological similarities with the ISC lineage in the mammalian small intestine [36–38]. ISCs self-renew and give rise to transient, non-dividing progenitor cells called EnteroBlasts (EBs) that are lineage-restricted (by Robo/Slit signaling and differential Notch signaling) to differentiate into either absorptive EnteroCytes (ECs) or secretory EnteroEndocrine (EEs) cells [36,37,39]. ISCs are the only dividing cells in the posterior midgut of *Drosophila* and their entry into a highly proliferative state is regulated by multiple stress and mitogenic signaling pathways, including Jun-N-terminal Kinase (JNK), Jak/Stat, Insulin, Wnt, and EGFR signaling [38,40].

During aging, flies develop epithelial dysplasia in the intestine, caused by excessive ISC proliferation and deficient differentiation of EBs [41,42]. This phenotype is a consequence of an inflammatory condition initiated by immune senescence and dysbiosis of the commensal bacteria, and causes metabolic decline, loss of epithelial barrier function, and increased mortality [43–45], and is associated with a strong tissue-wide increase in ER stress [12]. Increasing ER proteostasis in ISCs (by over-expressing Xbp1 or the ERAD-associated factor Hrd1) prevents the age-related over-proliferation of ISCs, suggesting that limiting ER stress-associated signaling in ISCs may be beneficial for tissue homeostasis [12].

Here, we have tested this hypothesis. We have explored the regulation of ISC proliferation by cell-autonomous and non-autonomous UPR^ER^ responses in detail, and have assessed the consequences of limiting ER stress responses in ISCs for longevity. By analyzing loss of function conditions for *Ero1L* we find that the induction of ISC proliferation by ER stress can be uncoupled from the production of ROS, but that ISC-specific activation of PERK is critical for the proliferative response. Interestingly, PERK activation in ISCs is triggered both by ER stress within ISCs and non-autonomously by ER stress in other cells of the intestinal epithelium, which activate PERK in ISCs through the secretion of Unpaired ligands and activation of JAK/Stat signaling in ISCs. PERK thus integrates epithelial stress responses to control ISC proliferation under challenging proteostatic conditions. Strikingly, PERK is also essential for normal cell proliferation in the ISC lineage, and excessive or chronic PERK activity in ISCs is a cause for the development of epithelial dysplasia in aging flies. Accordingly, we demonstrate that limiting PERK expression in ISCs is sufficient to extend lifespan.

## Results

### ROS-independent induction of ISC proliferation by ER stress

In a recent study we have shown that the control of ER proteostasis in ISCs by Xbp1 and Hrd1 (a key component of the ER-associated degradation pathway) is both sufficient and required to limit ISC proliferation. In Xbp1 or Hrd1 loss of function conditions, ER stress is associated with increased cellular ROS, and since ISC proliferation is stimulated by ROS [12,34,46], these results suggested that ROS production by the ER plays a critical role in the regulation of ISC proliferation during ER stress.

To test this notion further, we sought to uncouple ER stress from ROS production and disrupt ER homeostasis in ISCs by means that would not result in increased ROS production. Redox homeostasis of the ER is controlled by enzymes that promote disulfide bond formation and thus act as electron acceptors (including protein disulfide isomerase; PDI), and by ER oxidoreductins (including Ero1L) that transfer electrons from such enzymes to water, generating H_2_O_2_ [47]. Accordingly, in *Ero1L* loss of function conditions protein folding in the ER is perturbed, while the generation of H_2_O_2_ is reduced [47]. This provides a genetic condition in which to test whether the proliferative activity of ISCs can be influenced by ER stress in the absence of ROS production. RNAi-mediated knockdown of *Ero1L* in ISCs and EBs (using the ISC/EB driver esg::Gal4), or in ISCs only (combining esg::Gal4 with EB-specific inhibition of Gal4 by Su(H)Gbe-mediated expression of Gal80 [12]), resulted in a significant increase in ISC proliferation, confirming that ER stress promotes ISC activity (Fig 1C). As expected, this condition did not increase ROS levels in ISCs, as measured by Dihydroethidium (DHE) fluorescence (Fig 1C and 1D) [12,34]. Knocking down an unrelated ROS generating enzyme expressed in the gut, Duox, in ISCs/EBs did not induce ISC proliferation (S1C Fig). Conversely, over-expressing Ero1L exclusively in ISCs was sufficient to limit ISC proliferation in animals exposed to the ER stress inducer Tunicamycin (TM, which inhibits N-linked protein glycosylation and folding) (Fig 1E).

We confirmed the effect of Ero1L on ISC proliferation by generating MARCM clones from ISCs homozygous for the *Ero1L* loss of function allele *ero1l* ^*335qrs*^, or expressing Ero1L^RNAi^. Ero1L-deficient clones grew much faster than wild-type clones (Fig 1F), but this increase was accompanied by an accumulation of small, diploid, Dl positive cells, indicating that loss of *Ero1L* disrupts Notch-mediated differentiation of EBs (S1A Fig). Loss of ER homeostasis disrupts Notch signaling by preventing proper processing of the Notch receptor [48,49], and loss of Notch in EBs results in the formation of ISC ‘tumors’ consisting of symmetrically dividing, diploid Dl+ cells [36–38]. To confirm that ER stress promotes ISC proliferation in *Ero1L* loss of function conditions, we over-expressed spliced Xbp1 or CncC (both molecules improve ER homeostasis and influence ISC proliferation [12]). Indeed, this significantly limits over-proliferation of *Ero1L*-deficient ISCs (S1B Fig).

While these results demonstrated that ER stress can induce ISC proliferation independently of ROS production, it remained unclear whether mitotic activity of ISCs was directly stimulated by ER stress, or whether the increased number of mitotic ISCs in *Ero1L* loss of function conditions was a consequence of an increased rate of symmetric divisions due to the deficiency in Dl/N signaling. We therefore explored the activation of ER stress signaling pathways in Ero1L-deficient ISCs, aiming to identify potential signals that control mitotic activity. Loss of *Ero1L* resulted in increased eIF2α phosphorylation (Fig 1G), but did not increase Xbp1 expression (S1D Fig), suggesting that the PERK branch of the UPR^ER^ is selectively engaged. Knocking down PERK was sufficient to prevent eIF2α phosphorylation in *Ero1L* deficient ISCs (Fig 1H), confirming the specific requirement for PERK for this signal. Loss of PERK also prevented ISC proliferation in *Ero1L* deficient ISCs (Fig 1H and 1I), suggesting that PERK activity may promote ISC proliferation in response to ER protein stress independently of ROS production. To test this idea, we decided to explore the regulation of PERK activation in ISCs, and the role of PERK in the control of ISC proliferation in more detail.

### PERK is a central regulator of ISC proliferation

**Fig 2:**
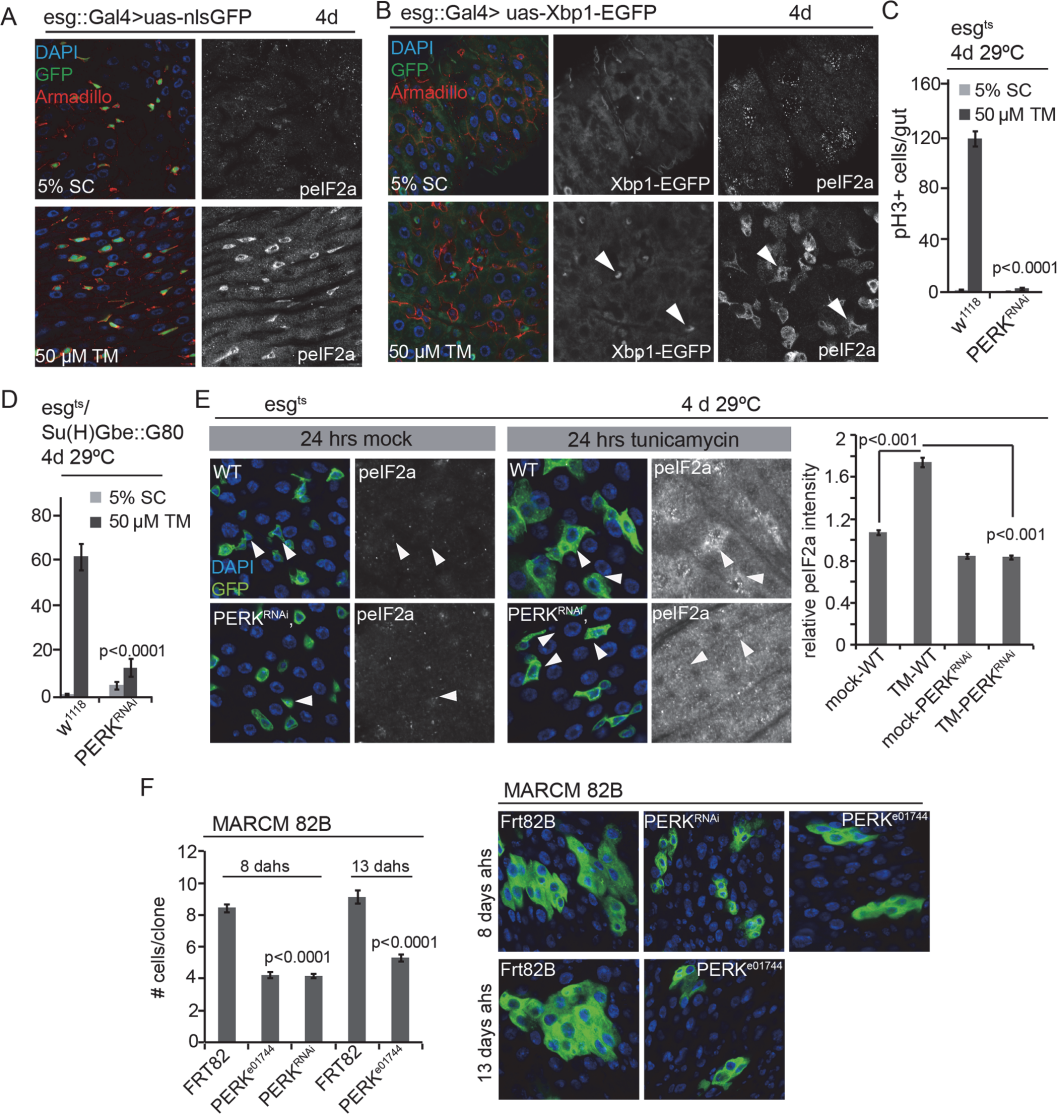
PERK integrates local and tissue-wide ER stress responses to regulate ISC proliferation. (A) Phosphorylation of eIF2α is induced in ISCs/EBs when young intestines are exposed to excessive ER stress. Guts of esg::Gal4, UAS::nlsGFP (nuclear-localized GFP) were immunostained with anti-peIF2α and anti-Armadillo antibodies in either the mock condition (5% sucrose) or after treatment with 50 μM Tunicamycin. (DNA: DAPI blue; GFP: green. Armadillo:red). peIF2α channel is shown separately on the right. (B) Xbp1 is spliced in ISCs/EBs when young intestines are exposed to excessive ER stress. GFP (GFP only expressed in Xbp1 splicing) and peIF2α channels are shown separately on the right. (C,D) Knockdown of PERK in ISCs/EBs (esg^ts^, C) or specifically in ISCs (esg^ts^/Su(H)Gbe::G80, D) prevents tunicamycin-induced ISC proliferation. Intestines of PERK^RNAi^ co-expressed with Dicer2 or wild-type (Dicer2 only) were examined. Averages and SEM are shown. P values from Student’s T test, N>10. (E) Phosphorylation of eIF2α in ISCs/EBs is not induced under ER stress in ISCs in which PERK is knocked down (esg^ts^). Intestines were exposed to either mock conditions (5% sucrose) or to 50 μM tunicamycin (TM). DAPI, blue; GFP, green, peIF2α shown as separate channel in white. Arrowheads for orientation. Quantification of phospho-eIF2α staining in ISCs relative to neighboring ECs in wild-type guts and in guts expressing PERK^RNAi^ in ISCs/EBs (esg^ts^) shown on the right. Averages and SEM are shown. P values from Student’s Test. (F) Quantification of MARCM clone sizes at 8 days and 13 days after heat shock for *PERK* loss-of-function (PERK^e01744^, PERK^RNAi^) conditions. Averages and SEM are shown. P values from Student’s T test. Number of clones examined: n = 455 (8 d FRT82B); n = 213 (8 d PERK^e01744^); n = 389 (8 d PERK^RNAi^); n = 200 (13 d FRT82B); n = 198 (13 d PERK^e01744^). Representative images for *PERK* loss-of-function (PERK^e01744^, PERK^RNAi^) at 8 days and 13 days after heat shock are shown on the right. (DAPI, blue; GFP, green). See also S2 and S4 Figs.

In a recent study we have shown that ISC-specific perturbation of ER proteostasis (by Xbp1 or Hrd1 knock-down) increases phosphorylation of eIF2α in ISCs [12] (S2A Fig). Increased eIF2α phosphorylation is also observed in ISCs/EBs after feeding flies the ER stress inducer Tunicamycin (TM, inhibiting N-linked protein glycosylation and folding) (Fig 2A), which also robustly induces ISC proliferation [12]. eIF2α phosphorylation in ISCs of TM treated flies is associated with increased Xbp1 splicing, a separate marker for ER stress, as determined by the expression of an Xbp1::GFP splicing reporter [50,51] (Fig 2B).

Strikingly, phospho-eIF2α (peIF2α) increased primarily in the progenitor cell population of flies exposed to Tunicamycin, suggesting that the PERK branch of the UPR^ER^ is activated specifically in ISCs and EBs even when ER stress is induced in a tissue-wide manner, and indicating that PERK activation has a specific role within ISCs in the regulation of the regenerative response to ER stress (Fig 2A and 2B). To test this idea, we assessed ISC proliferation in conditions in which PERK was knocked down in ISCs specifically (Fig 2C and 2D, RNAi was enhanced by co-expression of Dicer2). Knockdown of PERK in ISCs and EBs, or in ISCs specifically, was sufficient to inhibit TM-induced ISC proliferation (Fig 2C and 2D) and prevent TM-induced phosphorylation of eIF2α in ISCs (Figs 2C, 2D, 2E and S4A), confirming that PERK is required both for the phosphorylation of eIF2α and for the induction of ISC proliferation in these conditions. Knockdown of PERK in ECs, on the other hand, did not inhibit ISC proliferation, but stimulated their proliferation, similar to knockdown of ATF6 or Ire1 (S4B Fig).

We confirmed the role of PERK in ISC proliferation by assessing the growth of ISC lineages that were deficient in PERK, using mosaic analysis with a repressible cell marker (MARCM) to generate either ISCs homozygous for the PERK insertion allele *PERK* ^*e017*44^, or ISCs expressing dsRNA against PERK. In both conditions, clone growth was significantly delayed compared to wild-type controls, indicating that PERK activity is not only required for stress-induced ISC proliferation, but also for ISC proliferation during homeostatic regeneration (Fig 2F).

The impaired ISC proliferation in PERK loss of function conditions contrasts with the induction of ISC proliferation in Xbp1 or Hrd1 loss of function conditions [12], indicating that the primary function of PERK in ISCs, rather than promoting ER proteostasis (and thus limiting ISC proliferation) is to serve as a sensor of ER stress and an inducer of ISC proliferation. Accordingly, knockdown of PERK was also sufficient to limit ISC proliferation in other mitogenic conditions, such as proliferation induced either by over-expressing the JNK kinase Hemipterous (Hep) or by knocking down Notch (S2E and S2F Fig).

**Fig 3:**
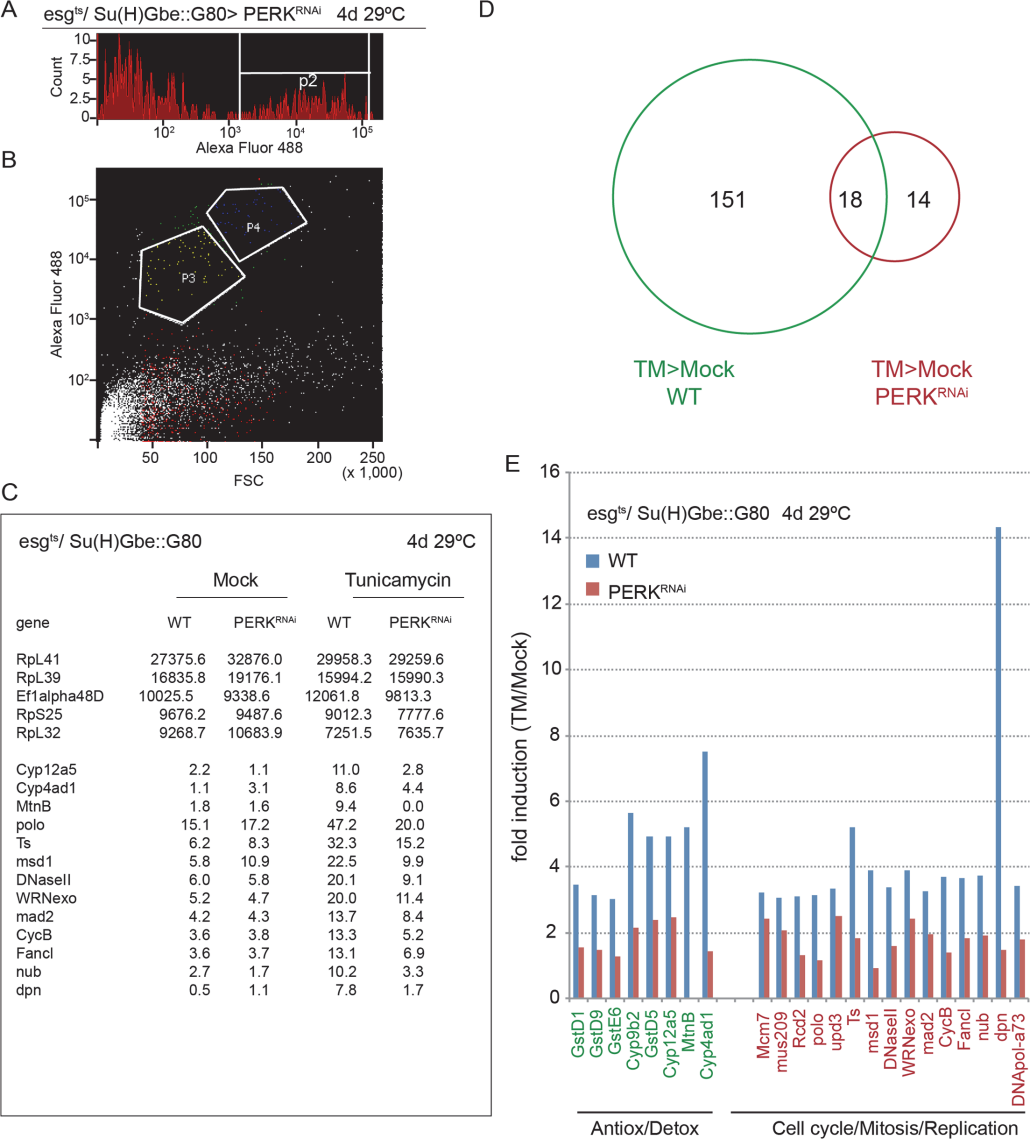
PERK-induced transcriptome changes in ISCs. (A, B) Cell sorting strategy to isolate ISCs from intestines expressing YFP in ISCs exclusively under the control of esg^ts^, Su(H)Gbe::Gal80. Cells were gated based on forward and side scatter, YFP fluorescence (A), and cell size (B). Gate P3 (ISCs) was sorted and collected for subsequent RNAseq analysis. Cells in P3 are Dl+ (Sousa-Victor, *personal communication)*. (C)FPKM values for selected genes across the four analyzed libraries (wild-type and PERK^RNAi^ expressing ISCs in both mock and tunicamycin-treated guts). A selected set of highly expressed genes is shown in the upper 5 rows, revealing no significant differences in expression across the four libraries. The lower rows show selected genes induced by Tunicamycin in wild-type ISCs, but not in PERK^RNAi^-expressing ISCs. Total cell numbers of ISCs isolated for RNAseq analysis are: mock WT (119,000), mock PERK (111,000), TM WT (112,000) and TM PERK (70,000). (D) Venn diagram for TM-induced transcriptional changes by RNA-seq analysis in wild-type ISCs and ISCs expressing dsRNA against PERK. Genes were considered induced when FPKM differences were at least 3 fold and FPKM values were at least 5.0. (E) Induction of genes involved in antioxidant and detoxification responses and in cell cycle regulation and mitosis in response to tunicamycin. Data was acquired by RNA-seq analysis of ISCs isolated by FACS. Fold induction (TM/Mock) is shown for wild-type and PERK-deficient ISCs. See details in Materials and Methods. See also S3 Fig.

Due to this unexpected function of PERK, and to characterize the ISC-specific PERK response, we decided to explore the transcriptome changes induced in ISCs by PERK using RNAseq. We isolated ISCs by FACS from wild-type guts expressing only YFP in ISCs (under the control of esg::Gal4 combined with Su(H)Gbe::Gal80) or from guts expressing YFP and PERK^RNAi^ using described protocols [52]. In both genotypes, we compared the transcriptomes of cells isolated from mock or TM treated flies (after 24 hours of TM exposure) using standard RNAseq procedures (Illumina MiSeq, [45]) (S1 Table). As expected, TM treatment resulted in a significant induction of genes involved in cell cycle, mitosis and DNA replication (as well as of genes involved in antioxidant and detoxification responses) in wild-type ISCs. In PERK-deficient ISCs, however, the induction of the vast majority (89%) of these genes was strongly reduced (Fig 3C–3E and S1 Table).

### Non-autonomous activation of PERK in ISCs by JAK/Stat signaling

**Fig 4:**
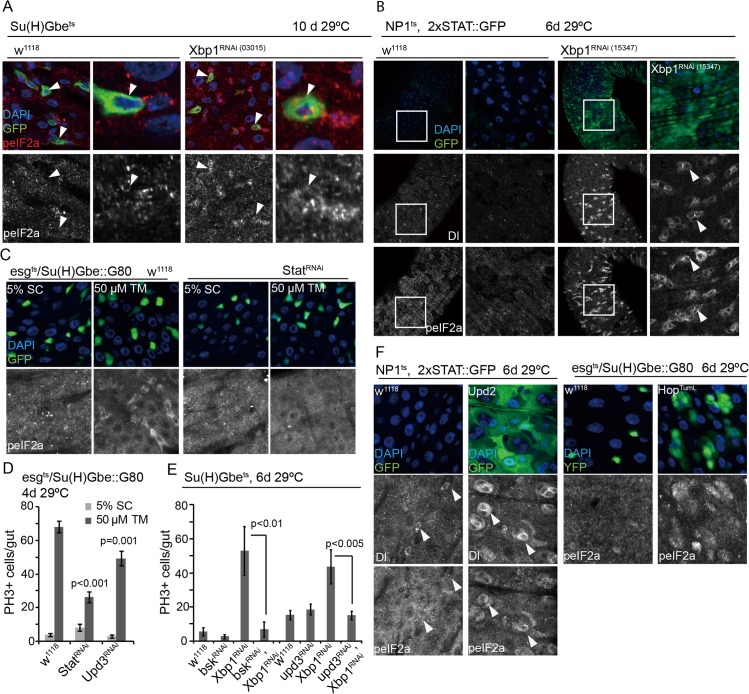
Non-autonomous activation of PERK in ISCs by JAK/Stat signaling. (A) Loss of Xbp1 (Xbp1^RNAi^: HMS03015) specifically in EBs (using Su(H)Gbe::Gal4,tubG80ts) increases the phosphorylation of eIF2α in ISCs/EBs. DAPI, blue; GFP, green, peIF2α red; peIF2α shown as separate channel in white. Arrowheads for orientation. (B) Increased phosphorylation of eIF2α in ISCs/EBs and activation of JAK/Stat signaling in the gut of flies in which Xbp1 is knocked down in ECs. 2XSTAT::GFP was used as a reporter for Stat activity. (DAPI, blue; GFP, green; Dl and peIF2α shown as separate channels in white). (C) Knockdown of Stat in ISCs (esg^ts^,Su(H)Gbe::G80) inhibits eIF2α phosphorylation in ISCs under ER stress. Intestines were exposed to either mock conditions (5% sucrose) or to 50 μM tunicamycin (TM). DAPI, blue; YFP, green, peIF2α shown as separate channel in white. Arrowheads for orientation. (D) Knockdown of Stat or Upd3 in ISCs prevents tunicamycin-induced ISC proliferation. Averages and SEM are shown. P values from Student’s T test, N>10. (E) Knockdown of JNK (Bsk^RNAi^) or Upd (Upd3^RNAi^) inhibits ISC over-proliferation induced by loss of Xbp1 in EBs (using Su(H)Gbe::Gal4,tubG80ts). Averages and SEM are shown. P values from Student’s T test, N>10. (F) Activation of JAK/Stat pathway either Upd2 overexpression in ECs (using NP1^ts^) or specifically in ISCs by over-exression of Hop^tuml^ (using esg^ts^/Su(H)Gbe::G80) promotes the phosphorylation of eIF2α in ISCs. DAPI, blue; GFP, green peIF2α and Dl shown as separate channel in white. See also S2, S4 and S5 Figs.

Our results indicated that ISCs initiate a regenerative response to both cell-autonomous as well as tissue-wide ER stress by activating PERK. We confirmed the notion of a non-autonomous control of PERK activity in ISCs by assessing the phosphorylation of eIF2α in ISCs of animals in which ER stress was induced specifically in EBs, ECs, fat body, or muscle. To perturb ER proteostasis in these cells and tissues, we knocked down Xbp1 (using Su(H)Gbe::Gal4, tub::G80^ts^ for EBs, NP1::Gal4, tub::Gal80^ts^ for ECs, ppl::Gal4, tub::G80^ts^ for fat body, and How::Gal4, tub::G80^ts^ for muscle). Knockdown of Xbp1 in EBs or ECs results in non-autonomous activation of ISC proliferation (Wang et al., 2014), and, consistent with an ISC-specific activation of PERK, also resulted in increased phosphorylation of eIF2α in ISCs (Figs 4A, 4B and S2B, note eIF2α phosphorylation in ISCs neighboring GFP—expressing EBs in Fig 4A, or in Dl+ ISCs in Fig 4B). However, knockdown of Xbp1 in fat body or muscle did not increase ISC proliferation or stimulate eIF2α phosphorylation in ISCs (S2C and S2D Fig), suggesting that the non-autonomous regulation of PERK is limited to cell/cell interactions within the intestinal epithelium.

Increased ER stress in intestinal epithelial cells has been associated with intestinal inflammation in vertebrates [4,6,7,10]. In flies, damage or stress in ECs promotes compensatory ISC proliferation by inducing inflammatory cytokines of the IL6 family, Unpaired 1–3 (Upd 1–3). These cytokines are induced in response to JNK activation in ECs and are secreted to activate JAK/Stat signaling in ISCs and in the surrounding visceral muscle [38,53,54].

To explore whether this compensatory proliferation program is involved in the non-autonomous regulation of ISC proliferation by ER stress, we asked whether loss of Xbp1 in EBs or ECs might influence ISC proliferation and ISC-specific PERK activity by stimulating JAK/Stat signaling. Consistent with a role for stress-induced Upd expression in the non-autonomous response to ER stress, loss of Xbp1 in ECs activates the Jak/Stat signaling pathway in muscle and epithelial cells of the gut (as determined using a reporter for Stat activity; 2XSTAT::GFP, [55] Fig 4B). Accordingly, knockdown of the JAK/Stat receptor Domeless, the JAK kinase Hop, or of Stat (but not of the JAK/Stat ligands Upd, Upd2, or Upd3) specifically in ISCs alleviated TM-induced ISC proliferation, and prevented phosphorylation of eIF2α in ISCs (Figs 4C and 4D and S4C–S4G). The induction of ISC proliferation by loss of Xbp1 in EBs could further be inhibited by knocking down the *Drosophila* JNK Basket (Bsk) or Upd3 (Figs 4E and S4H), confirming that JNK activation and Upd3 induction in EBs are required for the non-autonomous activation of ISC proliferation by ER stress in these cells.

To confirm that induction of Upd/JAK/Stat signaling is sufficient to activate PERK in ISCs, we determined the phosphorylation of eIF2α in ISCs over-expressing the activated form of the JAK Kinase Hopscotch (Hop^TumL^). When Hop^TumL^ was over-expressed in ISCs only (using esg::Gal4 combined with Su(H)::Gal80), eIF2α phosphorylation increased in labeled cells (Fig 4F, note that Hop^TumL^ induces the formation of clusters of labeled cells, suggesting an accumulation of ISCs and EB-like cells under these conditions). eIF2α phosphorylation also increased in ISCs when Hop^TumL^ was induced in ECs (using NP1::Gal4, tub::Gal80^ts^), suggesting a non-autonomous response of ISCs to JAK/Stat activation in neighboring ECs (S5B Fig). Similarly, over-expression of individual Upds (Upd, Upd2, Upd3) from ECs (using NP1::Gal4, tub::Gal80^ts^) stimulated eIF2α phosphorylation specifically in ISCs (Figs 4F and S5A). Taken together, these results provide a model for the non-autonomous control of ISC proliferation in response to ER stress in ECs: JNK-mediated induction of Upds from stressed ECs activates PERK via JAK/Stat signaling in ISCs, triggering ISC proliferation.

### Knockdown of PERK in ISCs extends lifespan

**Fig 5:**
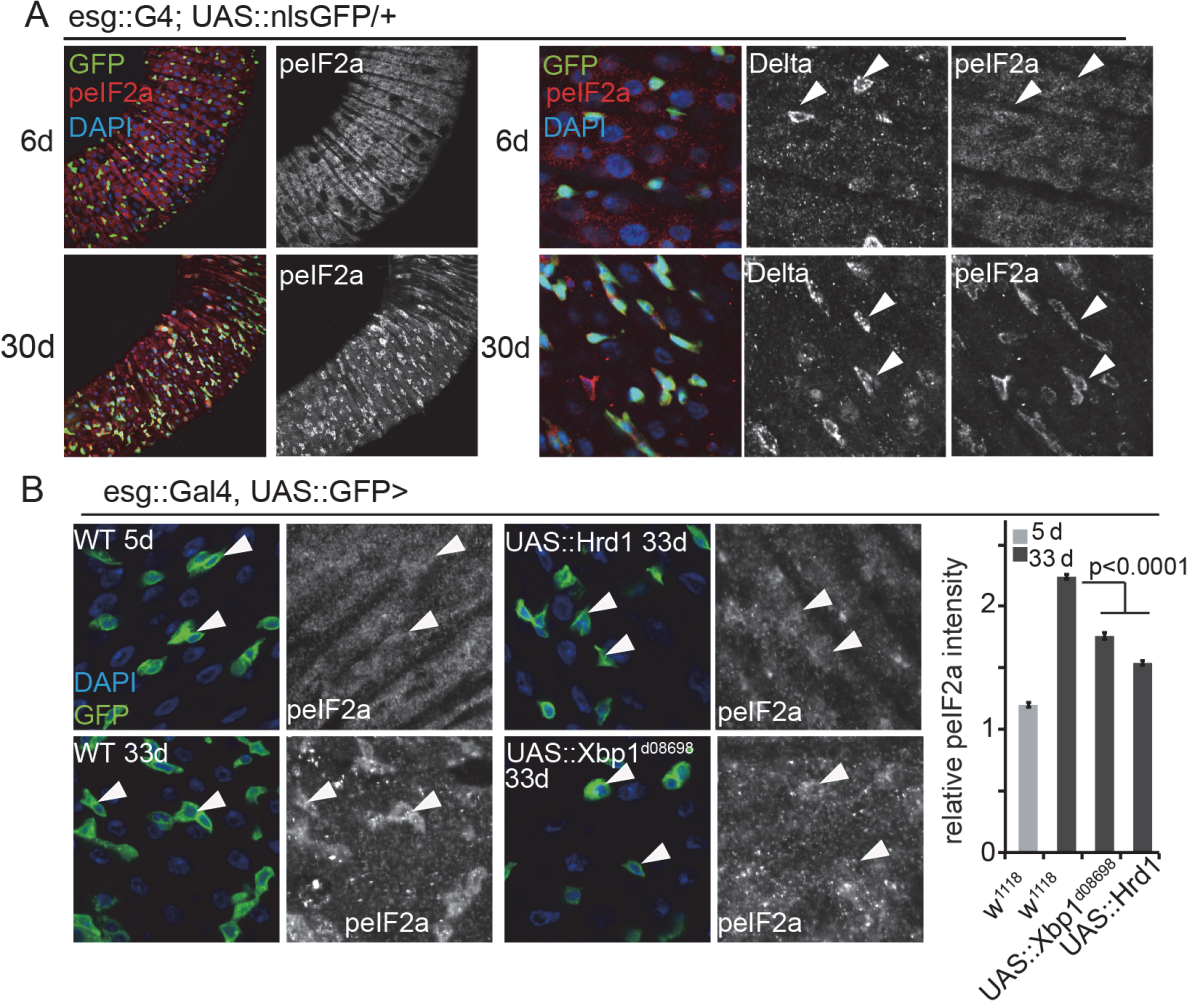
Limiting PERK activity in ISCs promotes intestinal homeostasis. (A) Young (5 day old) and old (30 day old) guts of esg::Gal4, UAS::nlsGFP flies immunostained with anti-peIF2α and anti-Dl antibodies. Enlarged imaged are shown on the right. White arrowheads point to individual ISCs (DNA: DAPI, blue; ISCs/EBs: GFP, green, peIF2α, red). peIF2α and Dl channels are shown separately on the right). (B) Intestines of wild-type flies stained with peIF2α antibodies at 5 days or 33 days of age and of old flies over-expressing Hrd1 or Xbp1 (Xbp1^d08698^) in ISCs/EBs (esg::Gal4) Arrowheads point to ISCs/EBs (marked by GFP). Relative peIF2α intensity was determined by calculating the ratio of fluorescence intensity in GFP+ cells and nearby ECs. Averages and SEM are shown. P values from Student’s T test, N > 160 (from 4–5 guts each).

The UPR^ER^ is broadly activated in the aging intestinal epithelium, and is associated with the development of age-associated dysplasia [12]. To assess the role of PERK activation in age-related ISC over-proliferation, we assessed the phosphorylation of eIF2α in the intestine of aging flies. Similar to the ISC-specific activation of PERK we observed in response to TM treatment, eIF2α phosphorylation was increased in aging intestines in an ISC-specific manner (Fig 5A). This activation was due to ER stress, as promoting ER homeostasis by over-expressing Xbp1 or Hrd1 (which maintains intestinal homeostasis by limiting age-associated ISC proliferation, [12]), was sufficient to limit the age-associated increase in eIF2α phosphorylation in ISCs/EBs (Fig 5B).

**Fig 6:**
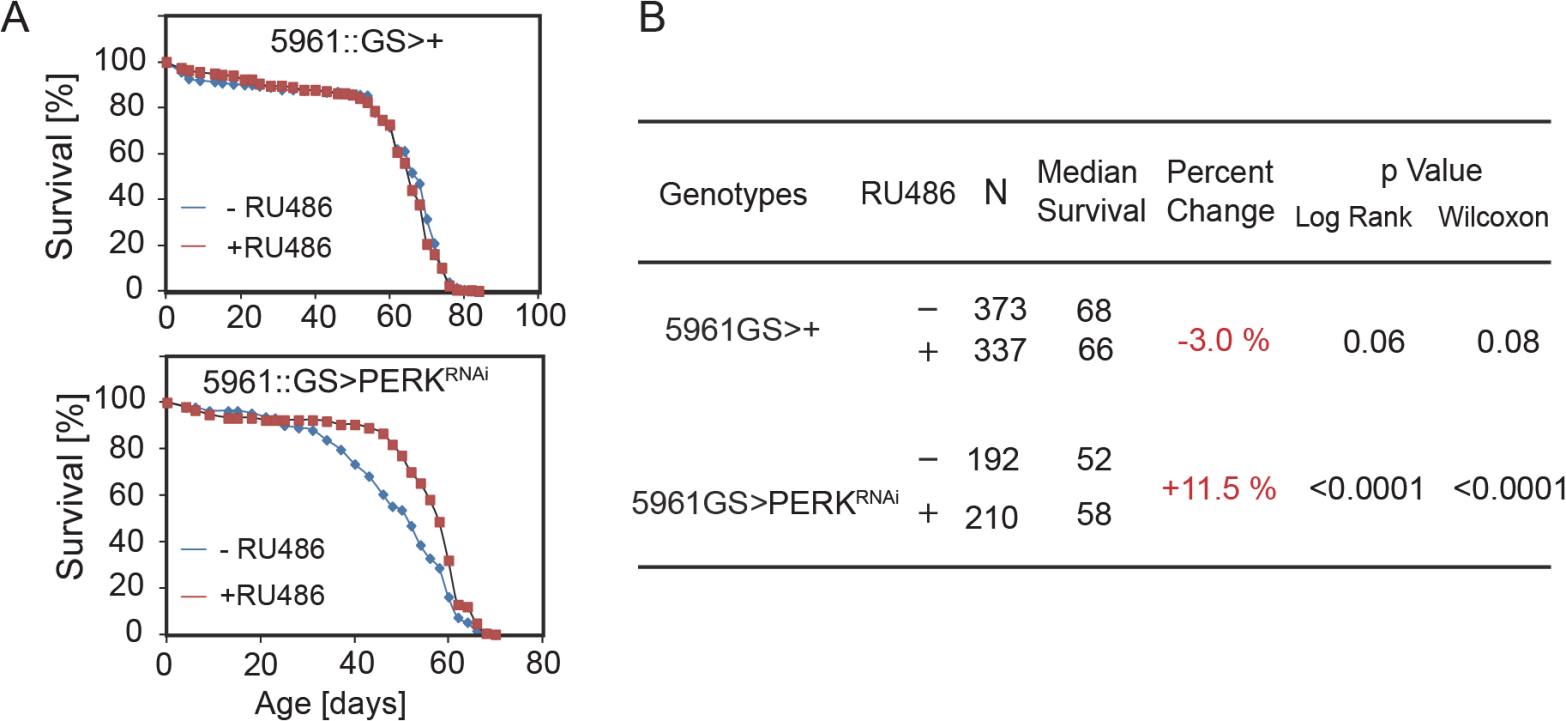
Knockdown of PERK in ISCs extends lifespan in flies. (A) Knockdown of PERK in ISCs using RU486-inducible driver 5961GS leads to lifespan extension. 200–400 female flies of each genotype were assayed. (B) Summary of parameters and lifespan statistics for the flies in Fig 6A. See also S6 Fig.

Since promoting proliferative homeostasis of the intestinal epithelium extends lifespan of flies (Biteau et al, 2010), we tested whether reducing PERK expression in ISCs was sufficient to extend lifespan. We used the RU486-inducible ISC/EB-specific 5961GS driver [44,56] to knock down PERK in ISCs and compare lifespan of genetically identical sibling populations. While RU486 treatment had no effect on lifespan of wild-type animals, RU486 treatment extended lifespan of animals expressing PERK^RNAi^ under the control of 5961GS (Fig 6A and 6B). Similarly, promoting ER homeostasis by over-expressing spliced Xbp1 in ISCs/EBs (using 5961GS) extends lifespan moderately (S6A Fig). Increased lifespan of flies expressing PERK^RNAi^ in ISCs was accompanied by improved barrier function of the intestine, as determined using a dye-penetration assay [43] (S6B Fig). The age-related activation of PERK in ISCs, which is a likely consequence of both tissue-wide and cell-autonomous ER stress, thus causes intestinal dysplasia, loss of barrier function of the intestine, and increased mortality in aging flies.

## Discussion

**Fig 7:**
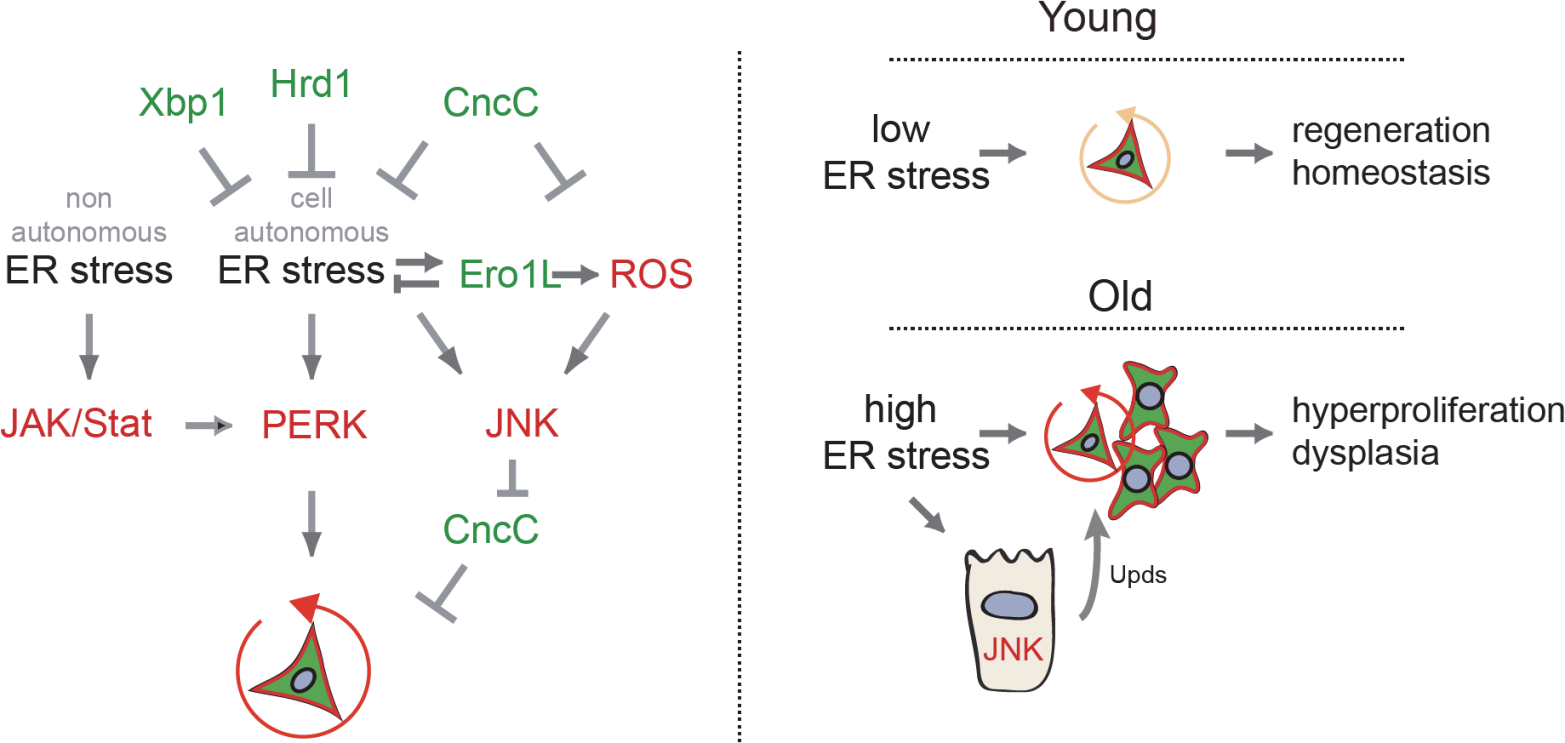
Model of UPR^ER^/ROS signaling network regulating ISC proliferation. Left: Model of UPR^ER^/ROS signaling network regulating ISC proliferation. PERK activation, either through cell-autonomous ER stress or in response to non-autonomous ER stress and JAK/Stat activation, is required for the induction of ISC proliferation. Inhibition of CncC activity by JNK in response to ER-induced ROS is further required to permit ISC regenerative responses. Right: Age-related loss of proliferative homeostasis as a consequence of increased ER stress.

Our results identify the PERK branch of the UPR^ER^ as a central node in the control of proliferative homeostasis in the intestinal epithelium, and establish a previously unrecognized role for PERK in promoting regenerative responses to both tissue-wide and cell-autonomous ER stress (Fig 7). This critical function of PERK in tissue regeneration, however, also results in the aging-associated loss of proliferative homeostasis in the intestinal epithelium, limiting organismal lifespan. The unique and specific increase in eIF2α phosphorylation in ISCs in stressed and aging conditions suggests a differential activation of the PERK-eIF2α branch of the UPR^ER^ between ISCs and their daughter cells. It remains unclear whether this differential regulation reflects different strategies in combating ER stress between these cell populations, and additional studies are necessary to address this interesting question.

### PERK and the integration of oxidative stress and ER stress responses

*Drosophila* ISCs, as many other stem cell types, are controlled extensively by redox signals [12,34,46]. Our previous work, as well as the results shown here, suggests that ER-induced oxidative stress plays a central role in the control of ISC proliferation after a proteostatic challenge. Our results support the notion that ER-induced ROS is a consequence of the PDI/Ero1L system, as has been proposed in mammalian cells [20]. However, Ero1L, as a thiol oxidase, may also affect the proper folding and maturation of Notch directly (as described previously [48]), inhibiting ISC differentiation, and resulting in stem cell tumors. The phenotype of Ero1L-deficient ISC lineages supports a role for Ero1L in Notch signaling (tumors with elevated numbers of Dl+ cells). At the same time, our results also support a role for Ero1L in limiting ISC proliferation directly through the UPR^ER^ (and independently of Notch signaling or oxidative signals), as loss of Ero1L induces PERK activity without promoting ROS production in these cells. PERK itself is required for the induction of cell cycle and DNA replication genes in ISCs responding to TM treatment, yet it also induces antioxidant genes under these conditions, suggesting complex crosstalk between PERK-mediated control of mitotic activity of ISCs and the control of redox homeostasis in these cells.

The fact that loss of *Ero1L* activates PERK while not inducing Xbp1 in ISCs suggests selective activation mechanisms for these two branches of the UPR^ER^. We propose that this selectivity is associated with the production of ROS and that ER protein stress activates the Xbp1 branch when associated with a ROS signal, while PERK can be activated by unfolded proteins independently of ROS production. Further studies are needed to dissect the relative contribution of ROS production, PERK activation and Notch perturbation in the control of ISC proliferation in *Ero1L* loss of function conditions.

### Non-autonomous control of PERK activity by JAK/Stat signaling

Our results highlight the interaction between cell-autonomous and non-autonomous events in the ER stress response of ISCs and support the notion that improving proteostasis by boosting ER folding capacity in stem cells improves long-term tissue homeostasis and can impact lifespan. The regulation of PERK activity in ISCs by the JAK/Stat signaling pathway provides a tentative mechanism for the interaction between IECs experiencing ER stress and ISCs: We propose that JNK-mediated release of JAK/Stat ligands from stressed IECs (as described in [54]) results in JAK/Stat mediated activation of PERK in ISCs, and that this activation is required for the proliferative response of ISCs to epithelial dysfunction. The activation of JAK/Stat signaling in the intestinal epithelium of animals in which Xbp1 is knocked down in ECs, the requirement for JNK activation and Upd expression in ECs for ISC proliferation in response to stress, and the requirement for Stat (and Hop and Dome) in ISCs for the activation of eIF2α phosphorylation and stress-induced ISC proliferation, support this model (Fig 7). The mechanisms by which Stat mediates activation of PERK remain unclear, and will be interesting topics of further study.

### PERK and age-related changes in ISC activity

Studies in worms have established the UPR^ER^ as a critical determinant of longevity, and Xbp1 extends lifespan by improving ER stress resistance [25,28]. Our data further support the notion that regulating ER stress response pathways is critical to increase health- and lifespan. Here, chronic PERK activation can be considered a downstream readout of the buildup of proteotoxic stress in the intestinal epithelium during aging, which then perturbs proliferative homeostasis by continuously providing pro-mitotic signals to ISCs. Knocking down PERK in ISCs limits these pro-mitotic signals, improving homeostasis and barrier function, and extending lifespan. Lifespan is generally extended when ISC proliferation is limited in older flies, but not when it is completely inhibited [34,44,45,57,58]. Accordingly, we observe lifespan extension when PERK is knocked down using an RNAi approach that does not completely ablate PERK function (note that experiments shown in Figs 1 and 2 were performed combining PERK^RNAi^ with Dicer2, which experiments in Fig 6 were performed using only PERK^RNAi^).

ER stress has been documented as tightly associated with intestinal inflammation and the development of IBDs in mice and humans [4,59,60]. Genetic variants in Xbp1 are associated with higher susceptibility to IBD [4] and a recent study indicates that Xbp1 can act as a tumor suppressor in the intestinal epithelium, by limiting intestinal proliferative responses and tumor development through the control of local inflammation [5]. In this context, the specific role of PERK in the control of ISC proliferation in the fly gut is consistent with the function of PERK in the intestinal epithelium of mice, where activation of PERK can promote transition of ISCs into the transient amplifying cell population [11]. While the *Drosophila* midgut epithelium does not contain a transit amplifying cell population, our data suggest that a role for PERK in the proliferative response of the ISC lineage to ER stress is conserved.

Due to the importance of the UPR^ER^ in the maintenance of tissue homeostasis in aging organisms, therapies targeting the UPR^ER^ are promising strategies to delay the aging process. Accordingly, pharmaceuticals that can limit ER stress (such as Tauroursodeoxycholic acid, TUDCA and 4-phenylbutyrate, PBA) have had therapeutic success in various human disorders [61,62]. Interestingly, flies fed PBA show increased lifespan, yet the effects of PBA on intestinal homeostasis have not yet been explored [63].

Studies from our lab and others highlight the importance of ISC function and proliferative homeostasis in fly longevity [34,44,45,58]. Based on this work, it is likely that further characterization of the effects of UPR^ER^-targeting drugs on ISC function and intestinal homeostasis will help develop clinically relevant strategies to limit human aging and extend healthspan.

## Materials and Methods

### Fly lines and husbandry

The following RNAi lines were obtained from the Vienna Drosophila RNAi Center: UAS::PERK^RNAi^ (v16427 and v110278), UAS::ATF6^RNAi^ (v36504), UAS::IRE1^RNAi^ (v39561), UAS::Xbp1^RNAi^ (v109312, v15347), UAS::Hrd1^RNAi^ (v6870), UAS::bsk^RNAi^, UAS::Ero1L^RNAi^ (v51169). Stat^RNAi^ (v106980), Domeless^RNAi^ (v106071), Hop^RNAi^(v40037), Upd^RNAi^. The following RNAi lines were obtained from the Bloomington *Drosophila* stock center: Upd^RNAi^ (33680), Upd2^RNAi^ (33949), Upd3^RNAi^ (32859)

Fly lines w^1118^, frt82B, UAS::nlsGFP, UAS::Xbp1^RNAi^ (TRip:HMS03015) were obtained from the Bloomington *Drosophila* stock center. The following fly lines were generously provided as indicated: *y1w1*; esg::Gal4/+ by Dr. S Hayashi; Su(H)Gbe::Gal4 by Dr. S. Bray; UAS::dEro1L and Ero1L^335qrs^ by Dr. H. Bellen; 2xSTAT-GFP by E.A. Bach;UAS::Upd2, UAS::Hop^tumL^ from David Bilder; UAS::Xbp1^d08698^ by Dr. P. Fernandez-Funez; UAS::Xbp1^spliced^ by Dr. P. Domingos; UAS::Upd by Dr. S.X.Hou; UAS:: Upd3 by Dr. N.Buchon; PEKR^RNAi^ combined with Dicer2 by Dr. S. Marciniak. *PERK* ^*e01744*^ is a Piggybac insertion line obtained from the Harvard Exelixis collection. According to the annotated information on Flybase, this line has a PBac{RB} element inserted into the 1^st^ intron of all three predicted PERK spliceforms, and exhibits recessive lethality.

All flies were raised on yeast/molasses-based food at 25°C and 65% humidity on a 12 hr light/dark cycle, unless otherwise noted.

For tunicamycin exposure, flies were starved in empty vials for 6–8 hrs and fed with 5% sucrose solution± 50μM tunicamycin for 24hrs followed by dissection in PBS.

For TARGET experiments, flies were raised at 18°C and shifted to 29°C at certain time points after eclosion. For MARCM clone induction, adult flies were aged for 1–2 days and then heat shocked at 37°C for 45 min.

All data were collected from female flies only.

### Immunostaining and microscopy

Guts were dissected in PBS, fixed for 45 min at room temperature in 100 mM glutamic acid, 25 mM KCl, 20 mM MgSO4, 4 mM sodium phosphate, 1 mM MgCl2, and 4%formaldehyde, washed for 1hr, and incubated with primary antibodies and second antibodies in washing buffer (PBS, 0.5% BSA, 0.1% Triton X-100).

The following primary antibodies were used: rabbit anti-peIF2α antibody (Cell Signaling: 3597, 1:150), rat anti-Delta (gift from Dr. MD Rand, University of Rochester, 1:1000); rabbit anti-pH3 (phosphorylated histone H3, Upstate, 1:1000), mouse anti-β-galactosidase (Developmental Studies Hybridoma Bank, 1:500), rabbit anti-β-galactosidase (Cappel, 1:5000), mouse anti-Armadillo (Developmental Studies Hybridoma Bank, 1: 250)

For Delta antibody staining, guts were fixed using a methanol-heptane method as descried (Lin et al., 2008).

Fluorescent secondary antibodies were purchased from Jackson ImmunoResearch Laboratories. DNA was stained using DAPI. Confocal imaging was performed on a Zeiss LSM700 confocal microscope and processed using ImageJ and Adobe Illustrator.

### ROS measurement via DHE

ROS levels were measured as described before (Hochmuth et al., 2011). Briefly, guts were dissected in Schneider’s medium, incubated in 30 μM (Invitrogen) for 5 min at room temperature in the dark, washed twice and mounted to be imaged immediately. GFP expressed under the control of esg::Gal4 or esg::Gal4, Su (H)::Gal80 was used to identify ISCs and/or EBs.

### Lifespan analysis

35 virgins (5961::GS homozygotes) were crossed to 20 *w* ^*1118*^; UAS::PERK^RNAi^(V16427), or spliced Xbp1 homozygous males. Progeny of these crosses was collected at 3 to 4 days after the first fly hatched. Flies were then separated according to sex and genotype, and females were placed into cages (50–80 flies/cage) and aged at 25°C. 100 μl of 5 mg/ml solution of RU486 or vehicle (80% ethanol) were added on the top of a food vial and dried overnight before fed to flies. Food was changed every other day. Demographic data were analyzed using Prism statistical software.

### FACS sorting and RNAseq

Wild-type Flies (esg^ts^,Su (H)GbeG80> w^1118^) and flies expressing dsRNA against PERK were exposed to 50μM tunicamycin and mock for 24 hrs (5% sucrose solution), followed by YFP+ labeled ISCs FACS sorting. Total RNA was then extracted using Trizol (Invitrogen) and used as template to generate RNA-seq libraries for Illumina sequencing. Expression was recorded as PRKM: reads per kbp per million reads.

## Supporting Information

S1 Fig Loss of Ero1L promotes ISC proliferation due to loss of ER homeostasis (related to Fig 1). (A) MARCM clones generated from *Ero1L* ^*335qrs*^ homozygous mutant ISCs. Arrowheads indicate examples of DI+ cells. (DAPI, blue; GFP, green; DI, Red). DI channel is shown separately on the right. (B) Over-expression of spliced Xbp1 or CncC inhibits ISC over-proliferation in *Ero1L* loss-of-function conditions (esg^ts^: esg::Gal4, tub::Gal80^ts^, UAS::GFP; DAPI, blue; GFP, green). Quantification of pH3+ cells is shown on the right. Averages and SEM are shown. P values from Student’s Test, N = 10. (C) eIF2α phosphorylation (left) and ISC proliferation (right) are not changed in DuoX-deficient ISCs/EBs (esg^ts^; DAPI, blue; GFP, green, peIF2α, white). Arrowheads for orientation. Averages and SEM are shown. P values from Student’s Test, N = 10. (D) Xbp1 expression (reporter line Xbp1p>Dsred, see also Wang et al., 2014) is unaffected when Ero1L is knocked down in ISCs/EBs (Arrowheads for orientation; esg^ts^; DAPI, blue; GFP, green, peIF2α).
(TIF)

S2 Fig Control of ISC proliferation by PERK (related to Figs 2 and 3). (A) Loss of Xbp1 induces eIF2α phosphorylation in ISCs/EBs. Xbp1 knockdown was achieved by expressing two different dsRNA constructs (Xbp1^RNAi109312^, Xbp1^RNAiHMS03015^) under the control of esg::Gal4,tubG80^ts^. DAPI, blue; GFP, green; peIF2α red; peIF2α channel shown separately in grayscale. Arrowheads for orientation. (B) Loss of Xbp1 (Xbp1^RNAi15347^) specifically in EBs (using Su(H)Gbe::Gal4,tubG80ts) increases eIF2α phosphorylation in ISCs and EBs. (C, D) Loss of Xbp1 (Xbp1^RNAi15347^) in fat body (using ppl::Gal4, tub::G80^ts^, C) or muscle (How::Gal4, tubG80^ts^, D) does not influence eIF2α phosphorylation in ISCs nor induce ISC proliferation. DAPI, blue; peIF2α or Dl shown as separate channels in white. Arrowheads for orientation. Averages and SEM are shown. P values from Student’s Test. (E) Knockdown of PERK inhibits ISC proliferation induced by Hep (JNKK) over-expression or by knock down of Notch in ISCs/EBs. DAPI, blue; GFP, green. GFP channel is shown separately on the left. (F) Quantification of pH3+ cells in the intestines of wild-type fly and flies expressing Hep or Notch^RNAi^ or co-expressing Hep or Notch^RNAi^ with PERK^RNAi^ in ISCs/EBs. Averages and SEM are shown. P values from Student’s Test, N = 10.
(TIF)

S3 Fig Analysis of sorted ISCs by FACS (related to Fig 3). Summarized parameters from sorting experiments of wild-type guts (esg^ts^,Su(H)Gbe::Gal80) and of guts expressing PERK^RNAi^ after mock or TM treatment.
(TIF)

S4 Fig JAK/Stat signaling mediates the non-autonomous regulation of PERK in ISCs (related to Figs 2 and 4). (A) eIF2α in ISCs/EBs is not phosphorylated under ER stress when PERK is knocked down in ISCs/EBs. Different fly lines expressing dsRNA against PERK in ISCs/EBs (using esg::Gal4, tubG80^ts^) were used. (DNA: DAPI blue; ISCs/EBs: GFP,green). Arrowheads point to selected ISCs/EBs. (B) Knockdown of PERK, ATF6 or IRE1 in Enterocytes (NP1::Gal4, tubG80^ts^) leads to epithelial dysplasia. Mitotic figures (pH3+ cells) were quantified along the whole gut. Averages and SEM are shown. P values from Student’s Test, N = 10. (C, D) Knockdown of Stat, Hop or Domeless in ISCs/EBs (esg^ts^, C) or specifically in ISCs (esg^ts^/Su(H)Gbe::G80, D) prevents tunicamycin-induced ISC proliferation. Averages and SEM are shown. P values from Student’s Test, N = 10. (E) Quantification of pH3+ cells in wild-type flies and flies expressing dsRNA against individual Upd ligands (Upd^RNAi33680^, Upd2^RNAi33949^ or Upd3^RNAi32859^) specifically in ISCs (esg^ts^/Su(H)Gbe::G80). Averages and SEM are shown. P values from Student’s Test, N = 10. (F) Knockdown of Stat, Domeless, Hop or Upd in ISCs (esg^ts^,Su(H)Gbe::G80) prevents eIF2α phosphorylation in ISCs in response to ER stress. Intestines were exposed to either mock conditions (5% sucrose) or to 50 μM tunicamycin (TM). DAPI, blue; YFP, green; peIF2α channel shown separately in grayscale. (G) Quantification of relative phospho-eIF2α staining in ISCs of wild-type guts and guts expressing STAT^RNAi^ in ISCs (esg^ts^, Su(H)Gbe::G80). Representative images shown in Fig 3C. Averages and SEM are shown. P values from Student’s Test. (H) Changes in ISC proliferation are not due to titration of Gal4 by multiple UAS constructs. Quantification of pH3+ cells in wild-type flies and flies expressing mcherry only, Xbp1^RNAi15347^ only, or co-expressing mcherry and Xbp1^RNAi15347^ specifically in EBs (Su(H)Gbe::Gal4,tubG80ts). Averages and SEM are shown. P values from Student’s Test, N = 10.
(TIF)

S5 Fig JAK/Stat nonautonomously regulates PERK activity in ISCs (related to Fig 4). (A) Overexpression of Upd or Upd3 in ECs (using NP1^ts^) induces phosphorylation of eIF2α in ISCs, accompanied with increased ISC proliferation (quantified by the frequency of pH3+ cells). DAPI, blue; peIF2α and Dl shown in grayscale. Averages and SEM are shown. P values from Student’s Test, N = 10. (B) Over-expression of Hop^TumL^ in ECs (using NP1^ts^) increases eIF2α phosphorylation in ISCs and promotes ISC proliferation. DAPI, blue; peIF2α and Dl in grayscale. Averages and SEM are shown. P values from Student’s Test, N = 10.
(TIF)

S6 Fig Modulation of UPR^ER^ signaling in ISCs influences lifespan in flies (related to Fig 6). (A) Over-expression of spliced Xbp1 in ISCs using the RU486-inducible driver 5961::GS results in moderate lifespan extension in flies (all female). Summary of parameters and lifespan statistics shown in the lower panel. (B) Knockdown PERK in ISCs/EBs (5961::GS) improves intestinal integrity in old flies. The percentage of “smurf” flies with / without RU486 at different ages is analyzed. N> 20.
(TIF)

S1 Table RNAseq data from sorted ISCs (related to Fig 2). Complete set of RNAseq results from sorted ISCs from wild-type flies exposed to sucrose (SCWT) or Tunicamycin (TMWT) or flies expressing PERK^RNAi^ exposed to sucrose (SCPK) or TM (TMPK). Data was generated using Illumina Miseq and mapped to release 5.45 of the *Drosophila* genome. Mapping and quantification was performed using the Tuxedo suite of bioinformatics tools.
(XLSX)
