# Supplementary figures and images for: PERK Limits Drosophila Lifespan by Promoting Intestinal Stem Cell Proliferation in Response to ER Stress
Source: PLoS Genet. 2015 May 6;11(5):e1005220. doi: 10.1371/journal.pgen.1005220 (PMC4422665; doi:10.1371/journal.pgen.1005220)

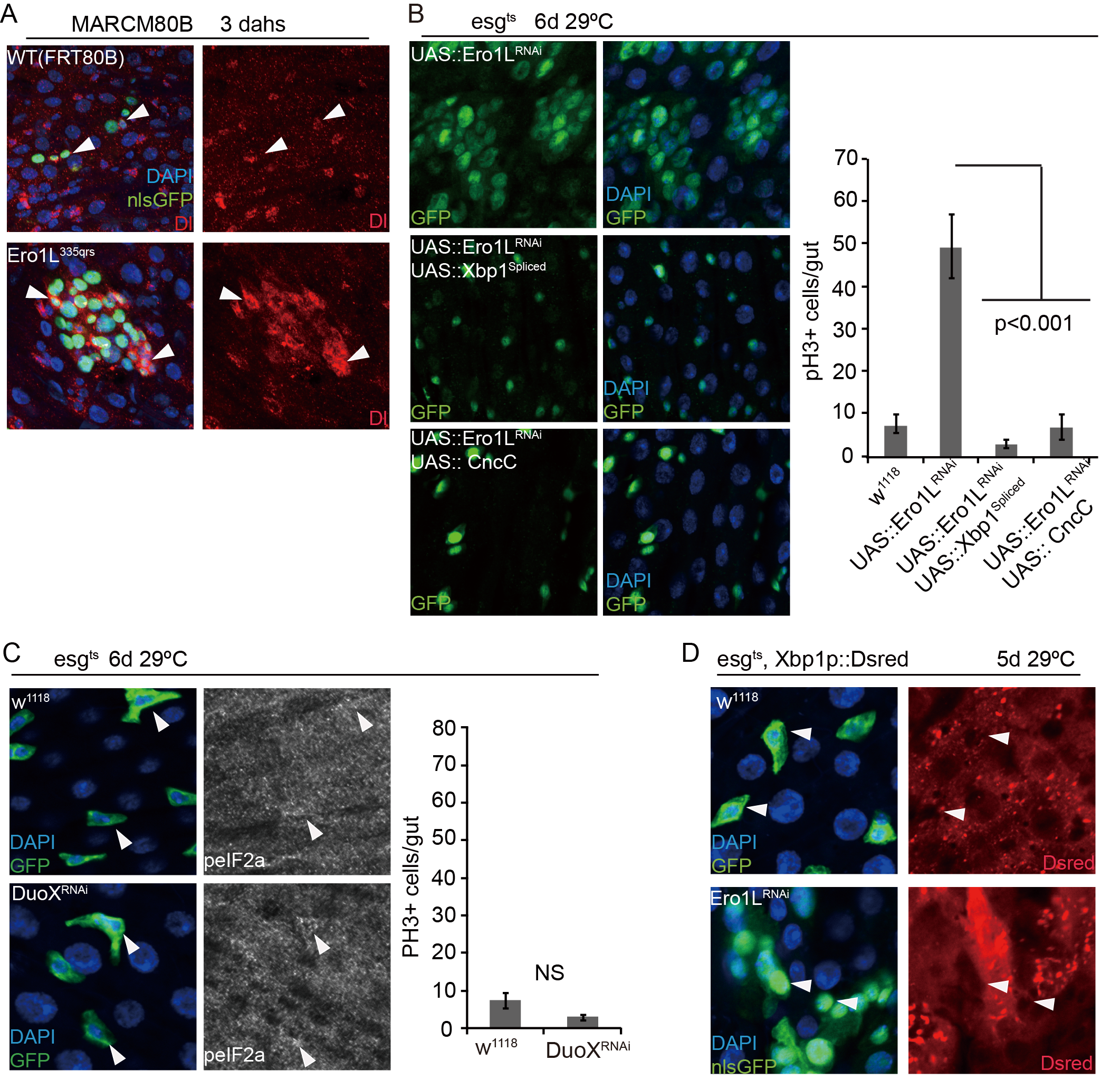

Supplement: S1 Fig — (A) MARCM clones generated from Ero1L 335qrs homozygous mutant ISCs. Arrowheads indicate examples of DI+ cells. (DAPI, blue; GFP, green; DI, Red). DI channel is shown separately on the right. (B) Over-expression of spliced Xbp1 or CncC inhibits ISC over-proliferation in Ero1L loss-of-function conditions (esgts: esg::Gal4, tub::Gal80ts, UAS::GFP; DAPI, blue; GFP, green). Quantification of pH3+ cells is shown on the right. Averages and SEM are shown. P values from Student’s Test, N = 10. (C) eIF2α phosphorylation (left) and ISC proliferation (right) are not changed in DuoX-deficient ISCs/EBs (esgts; DAPI, blue; GFP, green, peIF2α, white). Arrowheads for orientation. Averages and SEM are shown. P values from Student’s Test, N = 10. (D) Xbp1 expression (reporter line Xbp1p>Dsred, see also Wang et al., 2014) is unaffected when Ero1L is knocked down in ISCs/EBs (Arrowheads for orientation; esgts; DAPI, blue; GFP, green, peIF2α). (TIF) [file pgen.1005220.s001.tif]

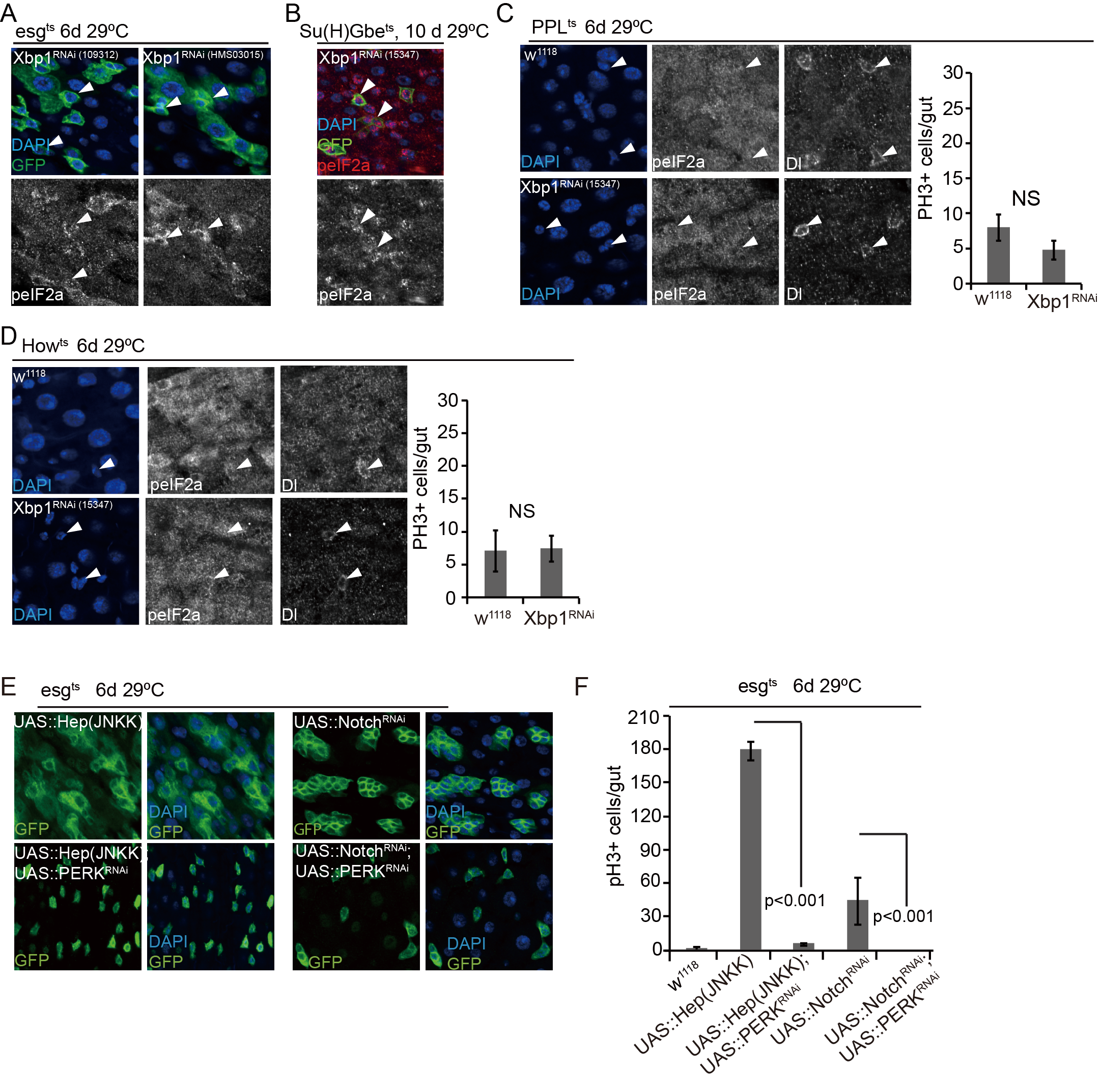

Supplement: S2 Fig — (A) Loss of Xbp1 induces eIF2α phosphorylation in ISCs/EBs. Xbp1 knockdown was achieved by expressing two different dsRNA constructs (Xbp1RNAi109312, Xbp1RNAiHMS03015) under the control of esg::Gal4,tubG80ts. DAPI, blue; GFP, green; peIF2α red; peIF2α channel shown separately in grayscale. Arrowheads for orientation. (B) Loss of Xbp1 (Xbp1RNAi15347) specifically in EBs (using Su(H)Gbe::Gal4,tubG80ts) increases eIF2α phosphorylation in ISCs and EBs. (C, D) Loss of Xbp1 (Xbp1RNAi15347) in fat body (using ppl::Gal4, tub::G80ts, C) or muscle (How::Gal4, tubG80ts, D) does not influence eIF2α phosphorylation in ISCs nor induce ISC proliferation. DAPI, blue; peIF2α or Dl shown as separate channels in white. Arrowheads for orientation. Averages and SEM are shown. P values from Student’s Test. (E) Knockdown of PERK inhibits ISC proliferation induced by Hep (JNKK) over-expression or by knock down of Notch in ISCs/EBs. DAPI, blue; GFP, green. GFP channel is shown separately on the left. (F) Quantification of pH3+ cells in the intestines of wild-type fly and flies expressing Hep or NotchRNAi or co-expressing Hep or NotchRNAi with PERKRNAi in ISCs/EBs. Averages and SEM are shown. P values from Student’s Test, N = 10. (TIF) [file pgen.1005220.s002.tif]

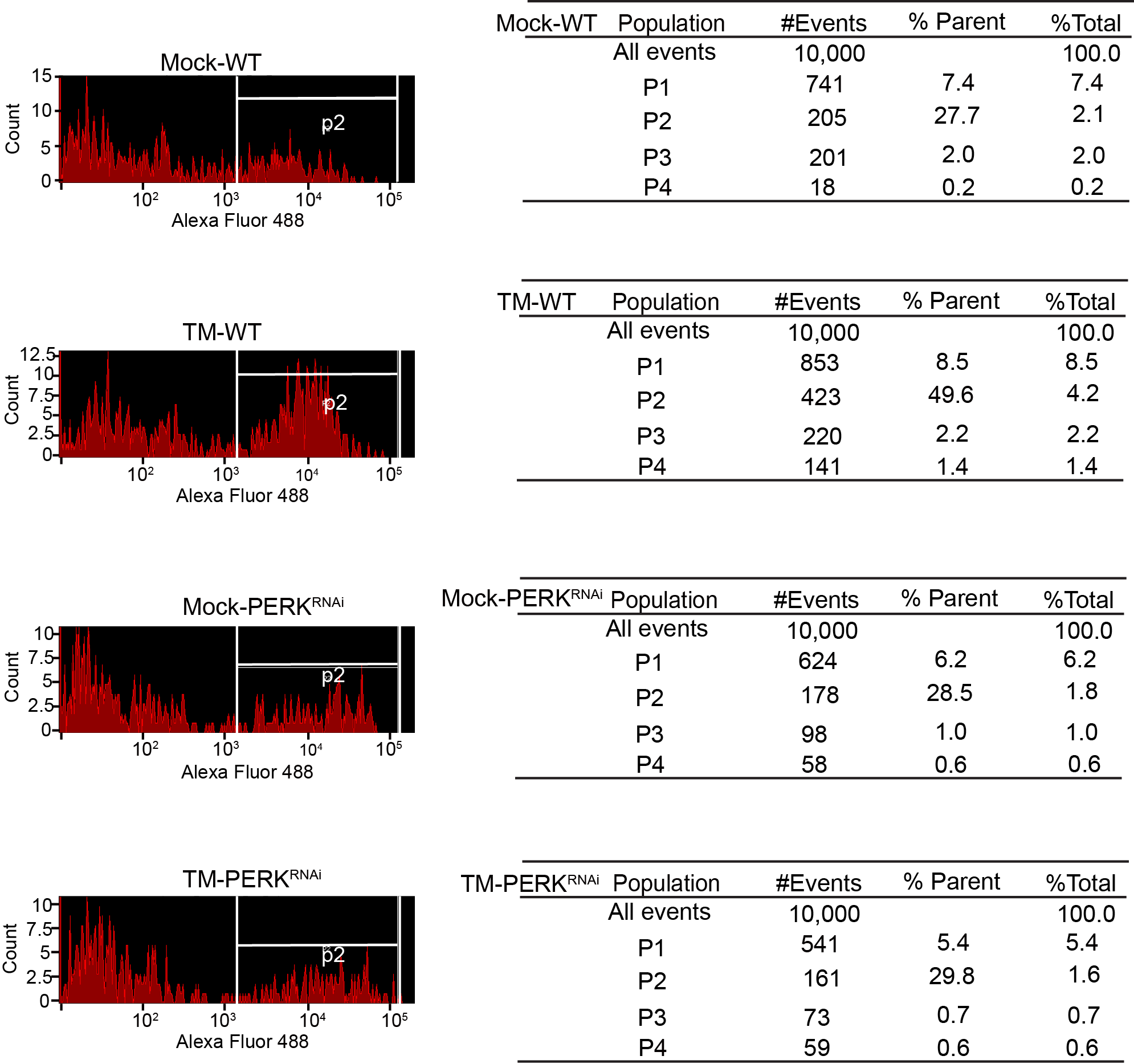

Supplement: S3 Fig — Summarized parameters from sorting experiments of wild-type guts (esgts,Su(H)Gbe::Gal80) and of guts expressing PERKRNAi after mock or TM treatment. (TIF) [file pgen.1005220.s003.tif]

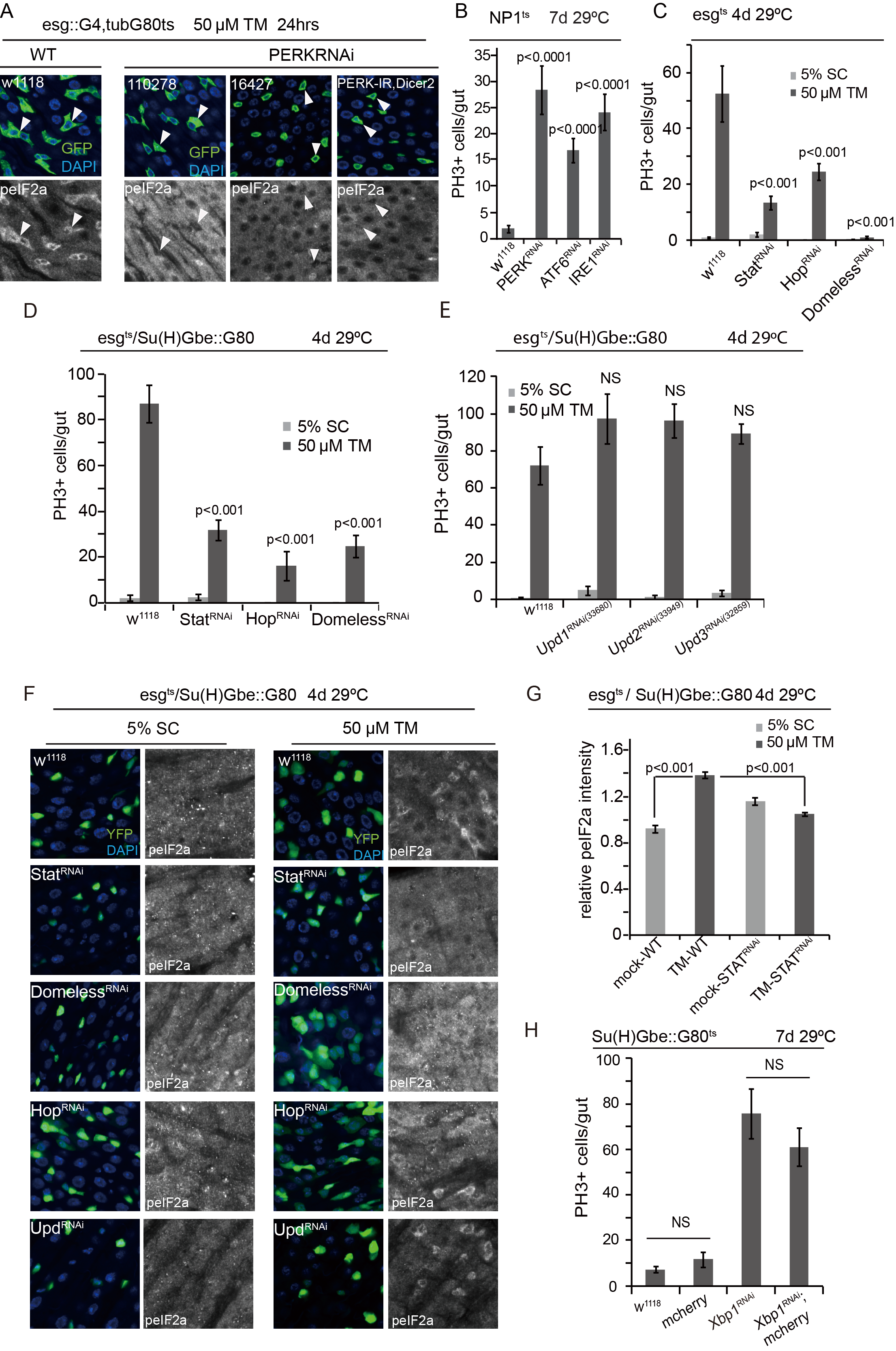

Supplement: S4 Fig — (A) eIF2α in ISCs/EBs is not phosphorylated under ER stress when PERK is knocked down in ISCs/EBs. Different fly lines expressing dsRNA against PERK in ISCs/EBs (using esg::Gal4, tubG80ts) were used. (DNA: DAPI blue; ISCs/EBs: GFP,green). Arrowheads point to selected ISCs/EBs. (B) Knockdown of PERK, ATF6 or IRE1 in Enterocytes (NP1::Gal4, tubG80ts) leads to epithelial dysplasia. Mitotic figures (pH3+ cells) were quantified along the whole gut. Averages and SEM are shown. P values from Student’s Test, N = 10. (C, D) Knockdown of Stat, Hop or Domeless in ISCs/EBs (esgts, C) or specifically in ISCs (esgts/Su(H)Gbe::G80, D) prevents tunicamycin-induced ISC proliferation. Averages and SEM are shown. P values from Student’s Test, N = 10. (E) Quantification of pH3+ cells in wild-type flies and flies expressing dsRNA against individual Upd ligands (UpdRNAi33680, Upd2RNAi33949 or Upd3RNAi32859) specifically in ISCs (esgts/Su(H)Gbe::G80). Averages and SEM are shown. P values from Student’s Test, N = 10. (F) Knockdown of Stat, Domeless, Hop or Upd in ISCs (esgts,Su(H)Gbe::G80) prevents eIF2α phosphorylation in ISCs in response to ER stress. Intestines were exposed to either mock conditions (5% sucrose) or to 50 μM tunicamycin (TM). DAPI, blue; YFP, green; peIF2α channel shown separately in grayscale. (G) Quantification of relative phospho-eIF2α staining in ISCs of wild-type guts and guts expressing STATRNAi in ISCs (esgts, Su(H)Gbe::G80). Representative images shown in Fig 3C. Averages and SEM are shown. P values from Student’s Test. (H) Changes in ISC proliferation are not due to titration of Gal4 by multiple UAS constructs. Quantification of pH3+ cells in wild-type flies and flies expressing mcherry only, Xbp1RNAi15347 only, or co-expressing mcherry and Xbp1RNAi15347 specifically in EBs (Su(H)Gbe::Gal4,tubG80ts). Averages and SEM are shown. P values from Student’s Test, N = 10. (TIF) [file pgen.1005220.s004.tif]

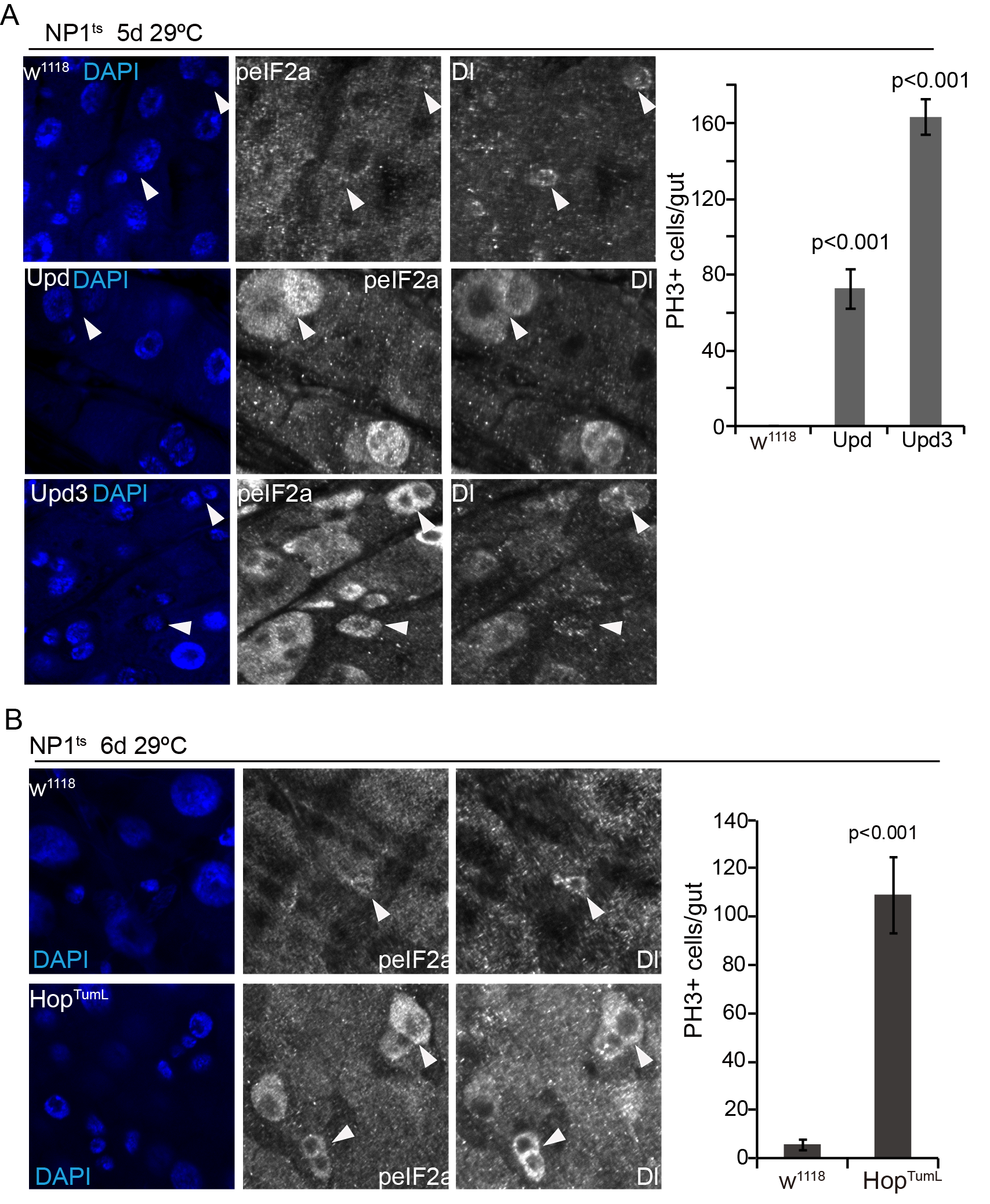

Supplement: S5 Fig — (A) Overexpression of Upd or Upd3 in ECs (using NP1ts) induces phosphorylation of eIF2α in ISCs, accompanied with increased ISC proliferation (quantified by the frequency of pH3+ cells). DAPI, blue; peIF2α and Dl shown in grayscale. Averages and SEM are shown. P values from Student’s Test, N = 10. (B) Over-expression of HopTumL in ECs (using NP1ts) increases eIF2α phosphorylation in ISCs and promotes ISC proliferation. DAPI, blue; peIF2α and Dl in grayscale. Averages and SEM are shown. P values from Student’s Test, N = 10. (TIF) [file pgen.1005220.s005.tif]

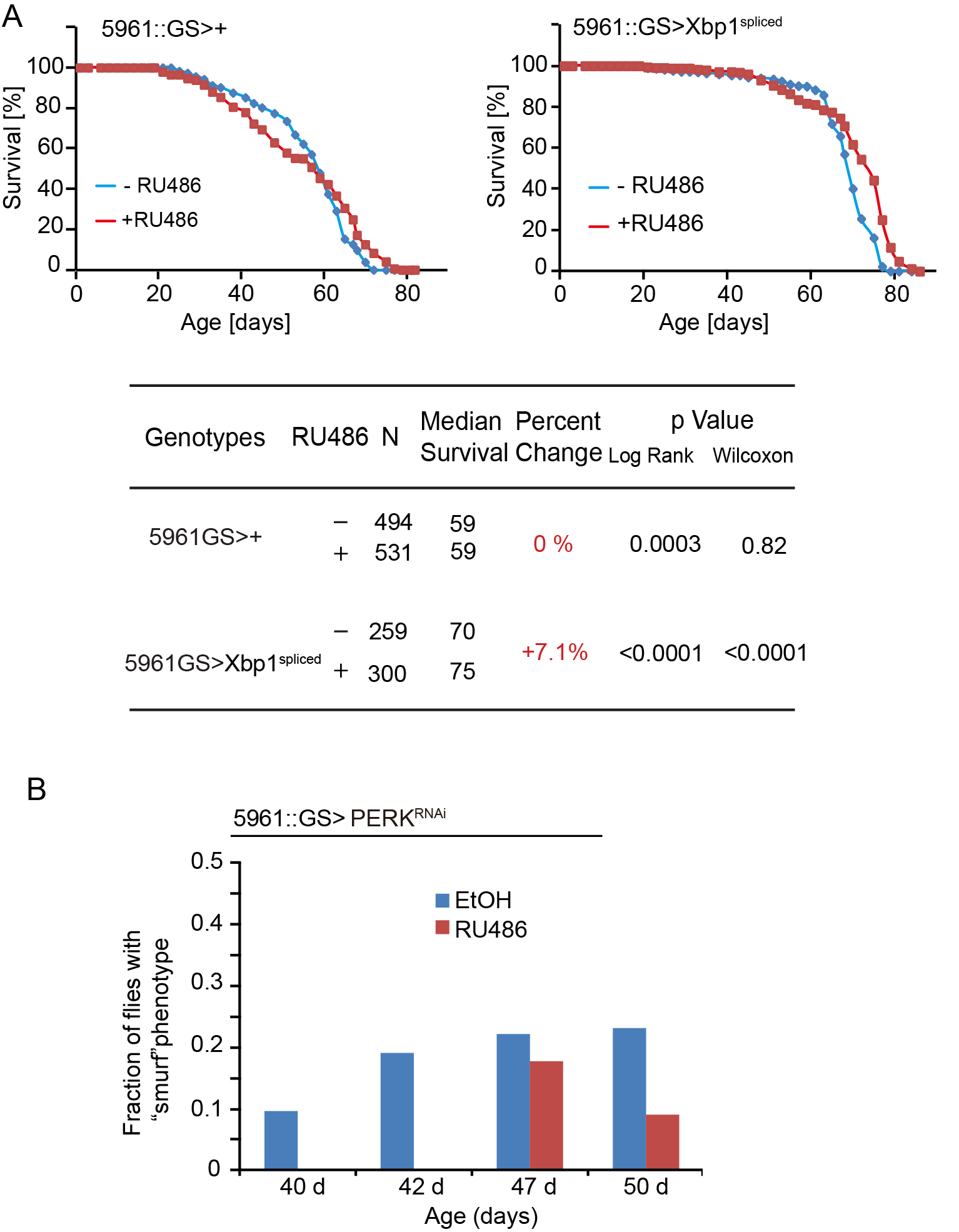

Supplement: S6 Fig — (A) Over-expression of spliced Xbp1 in ISCs using the RU486-inducible driver 5961::GS results in moderate lifespan extension in flies (all female). Summary of parameters and lifespan statistics shown in the lower panel. (B) Knockdown PERK in ISCs/EBs (5961::GS) improves intestinal integrity in old flies. The percentage of “smurf” flies with / without RU486 at different ages is analyzed. N> 20. (TIF) [file pgen.1005220.s006.tif]
